# ﻿*Gentianamopanshanensis* (Gentianaceae), a new species from Yunnan, southwest China

**DOI:** 10.3897/phytokeys.239.119800

**Published:** 2024-03-20

**Authors:** Tao Chen, Ting-Ting Wang, Shao-Yun Liu, Huan-Chong Wang

**Affiliations:** 1 School of Ecology and Environmental Science, Yunnan University, Kunming 650500, Yunnan, China Yunnan University Kunming China; 2 Herbarium of Yunnan University, Kunming 650091, Yunnan, China Herbarium of Yunnan University Kunming China

**Keywords:** Diffuse herbs, endemism, ITS sequence, Mopanshan Mountain, series *Fimbriatae*, Yunnan-Guizhou Plateau

## Abstract

*Gentianamopanshanensis*, a new species of the family Gentianaceae is here described and illustrated. This species is presently known only from the Mopanshan Mountain, Yunnan Province, southwest China. Phylogenetic analysis based on ITS sequence data has shown that this new species is a member of the series Fimbriatae of the section Chondrophyllae. Morphologically, it mostly resembles *G.mairei* and *G.panthaica*, but differs clearly from the latter two species in the shape and size of the leaves, and the characters of the corolla throat and plicae.

## ﻿Introduction

The genus *Gentiana* L. belongs to the family Gentianaceae and comprises more than 360 species that are distributed worldwide in the temperate and alpine regions, especially in Europe, Asia and North America (Ho and Liu 1990; Yuan et al. 1996; Yuan and Küpfer 1997). The plants of this genus are typical mountain plants, and most of the species are adapted to alpine habitats (Ho et al. 1996). The greatest diversity of *Gentiana* is found in the Tibeto-Himalayan region, where more than 250 species are native (Ho and Liu 2001). Relevant biogeographic studies have identified this region as the source area for *Gentiana* and related genera (Favre et al. 2016; Matuszak et al. 2016). The phylogenetic relationships within the genus *Gentiana* are now still incompletely clear, and its classification remains controversial (Favre et al. 2014, 2020; Sun et al. 2018; Sun and Fu 2019; Fu et al. 2021, 2022). Many *Gentiana* species have ornamental value and are also of pharmaceutical interest due to their interesting phytochemical properties (Mariana et al. 2013; Mirzaee et al. 2017; Jiang et al. 2021).

China is endowed with numerous species of *Gentiana*, including many endemics (Ho and Pringle 1995). While members of *Gentiana* can be found throughout the country, they are mostly concentrated in the Southwest Mountainous area, which includes the Qinghai-Tibet Plateau and Hengduan Mountains (Ho et al. 1988; Bai 2000). Ho and Pringle (1995) recognized 248 species from China in their "*Flora of China*", which accounts for more than 2/3 of the genus’ total species. Nevertheless, new species have been frequently discovered in China in recent years (e.g., Hsieh et al. 2007; Yang et al. 2008; Ho and Liu 2010; Wu et al. 2012; Yu et al. 2012; Yang et al. 2020; Favre et al. 2022). These new discoveries highlighted the need for continued field exploration and taxonomic research in this area.

During recent field surveys in Mopanshan Mountain, Xinping County, Yunnan Province, southwest China, we discovered an unknown species of *Gentiana*. After a phylogenetic analysis using ITS sequences to infer its systematic position and a detailed comparison with morphologically similar species, it became clear that this plant represents a distinct new species.

## ﻿Materials and methods

### ﻿Morphological analyses

This study including plant collection, specimen preparation, observation, and analysis followed the normal practice of plant taxonomic survey and herbarium taxonomy (Davis and Heywood 1963). Morphology of the new species was studied based on observation of living plants and specimens housed at YUKU. Digital images of type specimens of genus *Gentiana* are available at the JSTOR Global Plants (http://plants.jstor.org/) and at the Chinese Virtual Herbarium (http://www.cvh.ac.cn/); in addition, the collections housed at GBIF, KUN and YUKU were examined and compared with the new species. The dried specimens were examined under stereomicroscopes for morphological studies, and various organs were measured using rulers and metric vernier calipers. Terminology followed Ho and Liu (2001), Beentje (2010) and Mayfield (2021).

### ﻿Phylogenetic study

To determine the phylogenetic position of the putative new species, the internal transcribed spacer region (ITS) of the nuclear ribosomal DNA was used as the molecular marker. The total genomic DNA of this new species is extracted from silica-gel dried leaves using the DNA secure plant kit (Tiangen, Amsterdam, Netherlands). The PCR protocol followed by Shabir et al. (2022). The ITS primers used in this study were ITS4 and ITS5, as described by Yuan and Küpfer (1995) and He et al. (2016). The PCR products were bidirectionally sequenced with the same primers used for PCR amplifications in an ABI 3730 XL DNA Analyzer (Applied Biosystems) at the Kunming Branch of Beijing Qingke Biotechnology Co., Ltd. (Yunnan, China).

There are a total of 44 species of *Gentiana* to be used, which are representatives of most sections of *Gentiana*, including G.sect.Chondrophyllae Bunge, G.sect.Frigida Kusnezow, G.sect.Monopodiae (H. Smith) T. N. Ho, G.sect.Phyllocalyx T. N. Ho and G.sect.Stenogyne Franchet. In addition, *Swertiarosulata* (Baker) Klack, *Haleniataruga-gasso* Gilg and *Gentianellagentianoides* (Franchet) H. Smith were selected as outgroups. The dataset for phylogentic analysis consists of 60 taxa, 46 of which were obtained from the GenBank. Voucher specimen and GenBank accession information for taxon are listed in Appendix 1.

All sequences were aligned with MAFFT (Katoh and Standley 2013) using ‘auto’ strategy and normal alignment mode. Gap sites were removed with trimAl (Capella-Gutiérrez et al. 2009) using the “-automate” command. The best-fitting substitution models SYM+I+G model for Bayesian inference were selected using ModelFinder (Kalyaanamoorthy et al. 2017) in BIC criterion. MrBayes 3.2.6 (Ronquist et al. 2012) was used to conduct Bayesian phylogenetic analyses. Runs were performed for 5 million generations with a sampling of trees every 500 generations. The initial 25% of sampled data were discarded as burn-in.

## ﻿Results and taxonomic treatment

### ﻿ Gentiana mopanshanensis

Taxon classificationPlantaeGentianalesGentianaceae

Huan C. Wang & Tao Chen bis sp. nov.

86D24695-E975-53A0-8B74-0F809F99CD07

urn:lsid:ipni.org:names77338758-1

Figs 1 , 2 , 3

#### Type.

China. Yunnan Province: Xinping County, Mopanshan Mountain, near the top of mountain, alt. 2480 m, 23°56′23″N, 101°59′23″E, 3 April 2023, in flower, *H. C. Wang et al. XP19775* (Holotype: YUKU!; isotypes: YUKU!).

#### Diagnosis.

*Gentianamopanshanensis* is distinguishable from all other similar species of the genus by the combination of its rosulate basal leaves lanceolate to gladiate, up to 5 (6) cm long, cauline leaves lanceolate or linear-lanceolate, throat of corolla blue maculate, plicae with 5–10 fimbriations, and fimbriation irregular in length, usually 0.5–2 mm long.

**Figure 1.**
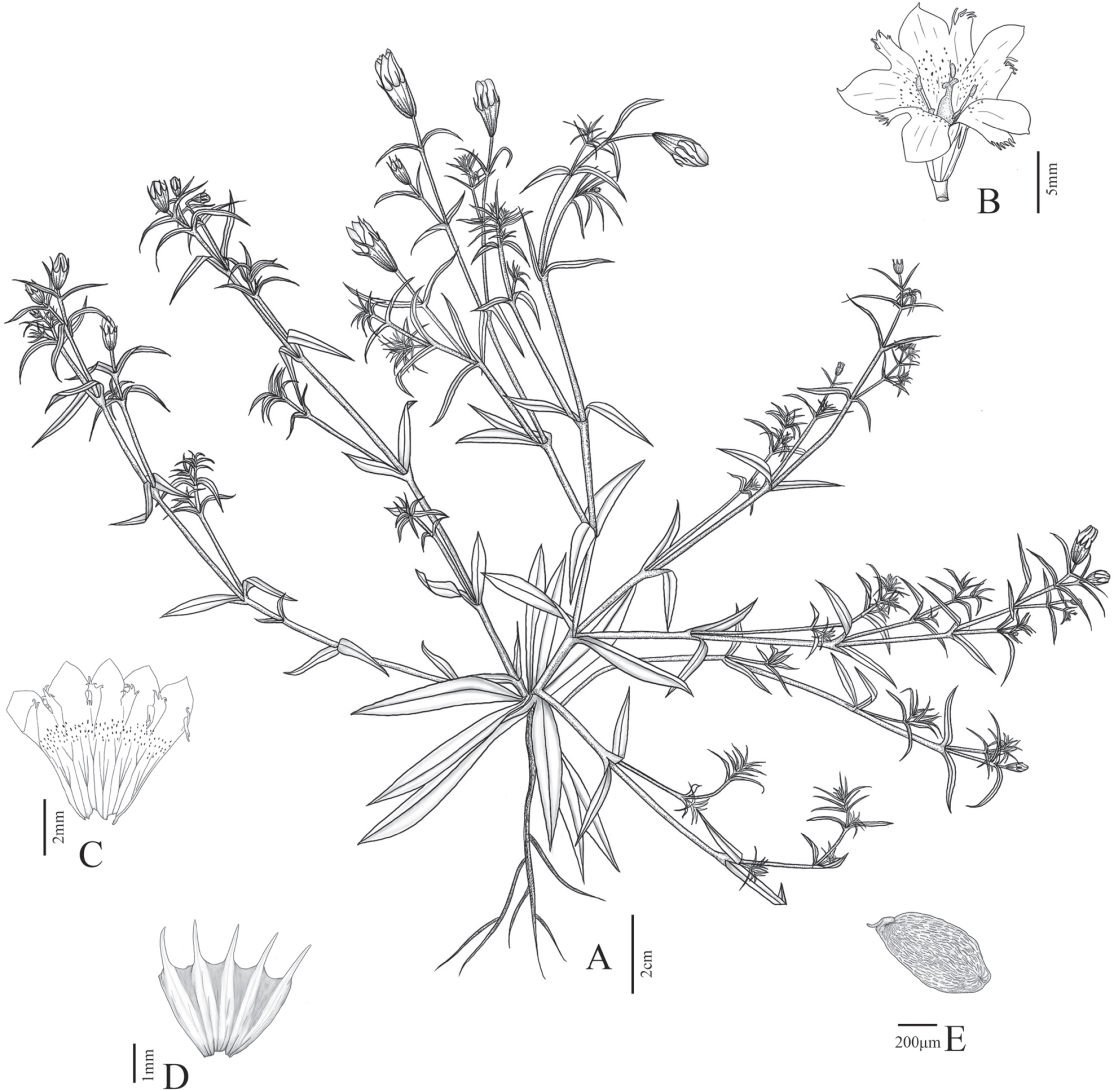
*Gentianamopanshanensis* sp. nov. (Drawn by Ting T. Wang) **A** habit **B** flower (front view) **C** dissected corolla **D** dissected calyx **E** seed.

#### Etymology.

The specific epithet “*mopanshanensis*” is derived from the type locality of the new species, the Mopanshan Mountain, and the Latin suffix -*ensis*, indicating the place of origin or growth.

**Figure 2.**
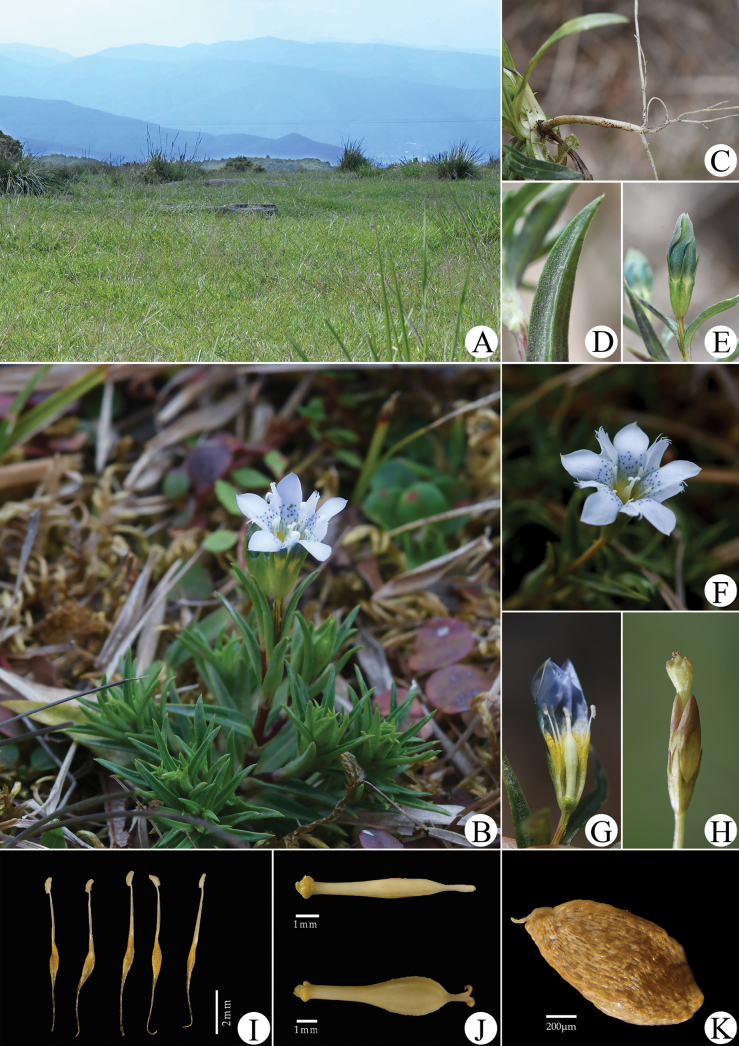
*Gentianamopanshanensis* sp. nov. (Photographed by HCW and TC) **A** habitat **B** habit **C** root **D** leaf blade (side view, showing adaxially densely and minutely papillate and transparent denticulate on margin) **E** closed flower **F** flower (front view) **G** dissected flower (showing the stamens and style) **H** mature fruit protruding from persistent corolla **I** stamens **J** pistils **K** seed.

#### Description.

Biennial herbs, diffuse, 5–15 cm in height. Root slightly fleshy, 4–6 cm long, with conspicuous rootstock. Stems yellow-green or purplish-red, smooth, much branched at base; branches procumbent or ascending. Basal leaves rosulate, persistent at anthesis, sessile or subsessile; blades lanceolate to gladiate, (1–) 3–5 (–6) cm long, 0.2–0.7 (–1) cm wide, both surfaces densely and minutely papillate, apex acuminate, margin transparent, densely denticulate, basal veins 1–3, distinct, midvein convex beneath. Cauline leaves opposite, semiamplexicaul, base proximally compounded, petiole tube 1–1.5 mm long; blades lanceolate or linear-lanceolate, 0.3–2 cm long, 0.1–0.6 cm wide, apex acuminate, margin transparent, densely denticulate, both surfaces densely and minutely papillate, basal veins 1–3, midvein convex beneath. Flowers solitary, terminal on branch. Pedicels yellowish green, glabrous, 3–12 mm long, purplish-red striped. Calyx 5–8 mm long, obconic, yellow-green; tube campanulate, 4–5 mm long, 2–3 mm in diameter, slightly longer than lobes; lobes 5, acicular or subulate, 1.5–3 mm long, papillate on margin; veins ridged on abaxial surface, decurrent towards calyx tube; sinus between lobes obtuse to sub-rounded. Corolla 8–14 mm long, 5–8 mm in diameter, trumpet-shaped, blue-white, outside with copper-green stripes, inside with deeply blue spots in throat; tube tubular, 5–8 mm long, 4–6 mm in diameter, golden inside; lobes ovate to broadly ovate, 2–3 mm long, 2–3 mm wide, apex acute, margin entire; plicae triangular-ovate, 2–2.5 mm long, apex irregularly laciniate, fimbriate, with 5–10 fimbriations, usually 0.5–2 mm long. Stamens 5, filaments filiform-subulate, 3–6 mm long, inflated at middle, inserted in lower middle of corolla tube; anthers rectangular-rounded, 0.6–1 mm long. Ovary stipitate, ellipsoid or fusiform, 2.5–3 mm long, apex obtuse, base attenuate; style clavate, 0.5–1 mm long, stigma bifid, extrorse. Capsules obovate, exerted beyond persistent corolla, gynophore up to 15 mm long, narrowly winged on both margins. Seeds ovate-triangular, up to 1.5 mm long, yellowish-brown, densely striato-reticulate on seed coats.

**Figure 3.**
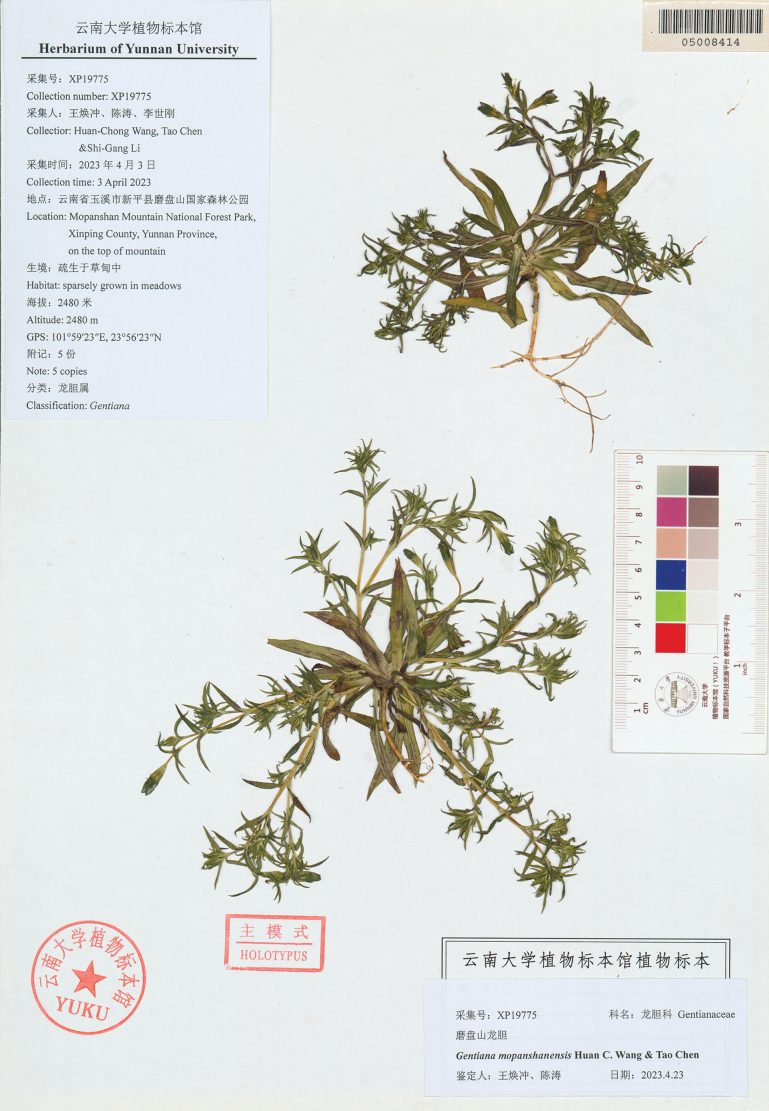
Holotype of *Gentianamopanshanensis* sp. nov.(YUKU-05008414).

#### Phenology.

*Gentianamopanshanensis* has a long flowering and fruiting period. It starts flowering in March and continues until August, and its fruiting period is from May to September.

#### Distribution and habitat.

**Figure 4.**
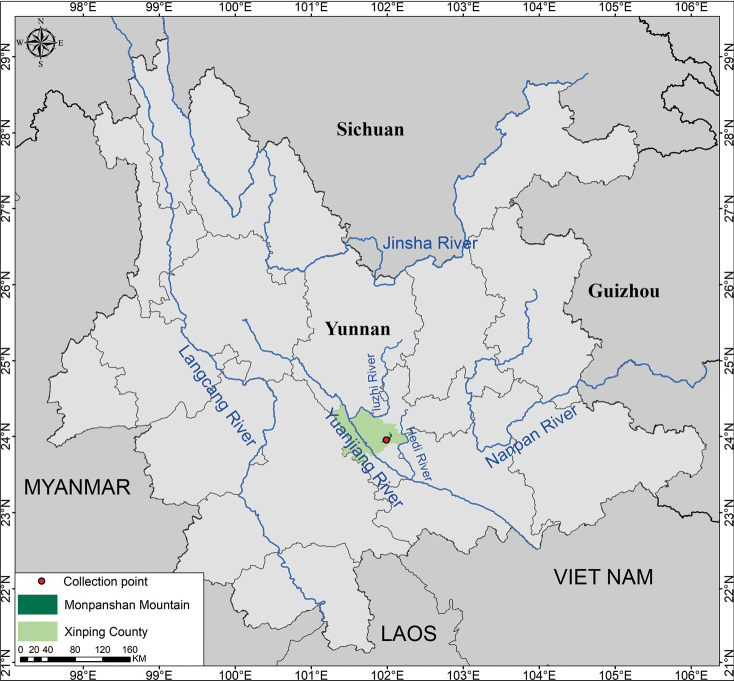
Geographical distribution of *Gentianamopanshanensis* sp. nov. (red dot).

*Gentianamopanshanensis* is currently only found in the Mopanshan Mountain (Fig. 4), which is located in the southeast of Xinping County, at the southwestern edge of the Yunnan-Guizhou Plateau. The mountain is situated east of the Yuanjiang River valley and has an elevation ranging from 1370 to 2611 meters. *G.mopanshanensis* usually occurs at elevations between 2400 and 2550 meters and mainly grows in wet meadows near the peak of the mountain. It can also be occasionally found under the thickets predominated by *Lithocarpusvariolosus* Chun (Fagaceae) and *Quercusguyavifolia* H.Lév. (Fagaceae). In meadow habitats, this new species is commonly associated with *G.praticola* Franchet (Gentianaceae), *Polygaladunniana* H.Lév. (Polygalaceae), *Arundinellahookeri* Munro ex Keng (Gramineae), *Fragarianilgerrensis* Schlecht. ex J. Gay (Rosaceae), *Roscoeatibetica* Batalin (Zingiberaceae) and *Bistortapaleacea* Yonek. et H.Ohashi (Polygonaceae).

#### Molecular phylogenetics.

The ITS sequence region of *Gentianamopanshanensis* comprises 625 base pairs with a GC content of 57.12%. The alignment of 60 ITS sequences resulted in a matrix of 662 total characters, of which 344 are constant, 93 of the variable characters are singleton sites and 225 characters are parsimony informative sites.

**Figure 5.**
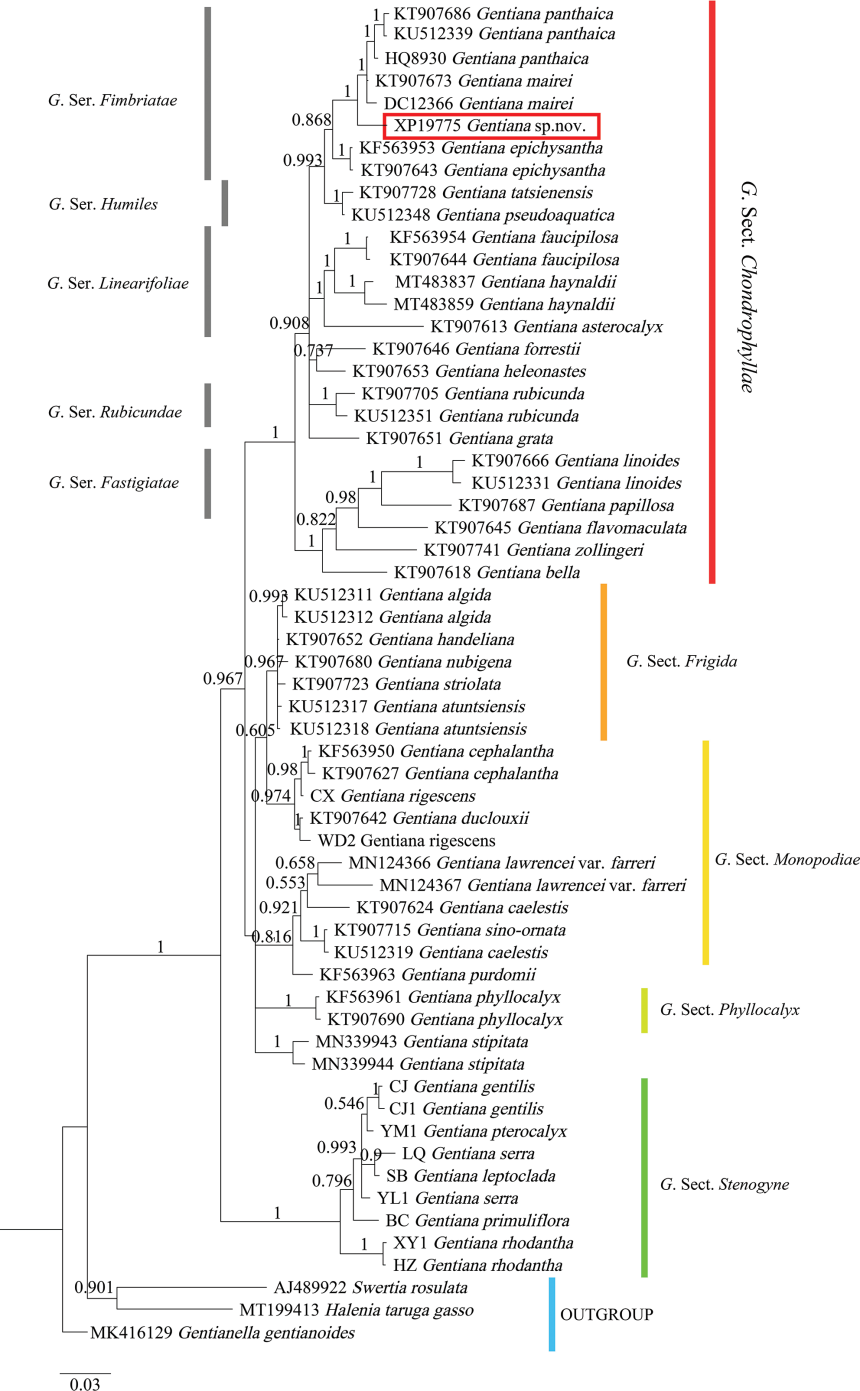
Mrbayes tree of *Gentiana* based on ITS sequences showing phylogenetic placement of *G.mopanshanensis*. Mrbayes posterior probabilities are shown near the nodes. *G.mopanshanensis* is marked by a red box.

As shown in the phylogenetic tree (Fig. 5), phylogenetic analyses using the ITS sequence data demonstrated that the new species belongs to a clade representing the GentianasectionChondrophyllae with maximum support. In this clade, *G.mopanshanensis* falls within the subclade corresponding to series *Fimbriata* Marq. with 0.993 posterior probabilities. It constituted a monophyletic lineage with *G.panthaica* Prain et Burkill and *G.mairei* H.Lév. with maximum support (PP = 1) and were resolved as sister to them. The phylogenetic result is also supported by the morphological characteristics.

#### Discussion.

Based on phylogenetic analyses, *Gentianamopanshanensis* should be assigned to the series Fimbriatae of the section Chondrophyllae. Its placement within this series is also supported by its particular morphological characters: stems much branched at the base, basal leaves well developed, leaf blades and calyx with densely and minutely papillate, calyx lobes acicular or subulate, plicae apex fimbriate, obovate capsule with strong and broad wings at apex.

This new species is most similar to *Gentianamairei* and *G.panthaica* in terms of habit, and flower shape and size, especially plicae apex fimbriate. However, *G.mopanshanensis* can be distinguished from *G.mairei* by its biennial (vs. annual) habit, basal leaves lanceolate to gladiate (vs. ovate to ovate-elliptic), (1–) 3–5 (–6) cm (vs. 0.7–1.4 cm) long, 0.2–1 cm (vs. 0.3–0.7 cm) wide, cauline leaves lanceolate or linear-lanceolate (vs. ovate-triangular to ovate-lanceolate), with blue spots (vs. blackish, white or pale yellow stripes) in throat, plicae with 5–10 (vs. 8–10) fimbriations, irregular (vs. nearly equal) in length, ususally 0.5–2 mm (vs. 2–2.5 mm) long. *G.mopanshanensis* differs from *G.panthaica* in biennial (vs. annual) habit, basal leaves lanceolate to gladiate (vs. ovate-elliptic to ovate), cauline leaves lanceolate or linear-lanceolate (vs. lanceolate, narrowly elliptic or ovate-triangular), plicae with 5–10 (vs. 15–20) fimbriations, filament laciniate (vs. filiform, curly). A detailed morphological comparison between these three species is summarized in Table 1.

**Table 1.** Morphological comparison of *G.mopanshanensis*, *G.maire*, *G.panthaica*.

Characters	*G.mopanshanensis*	*G.mairei*	*G.panthaica*
Habit	biennial	annual	annual
Leaf			
-Basal leaf	lanceolate to gladiate 3–5 (6) cm × 0.2–0.7 (1) cm	ovate to ovate-elliptic, 7–1.5 cm × 3–0.7 cm	ovate-elliptic to ovate, 0.9–2 cm × 0.4–1 cm
-Cauline leaf	lanceolate or linear-lanceolate, 0.3–1.5 cm × 0.1–0.4 cm	ovate-triangular to ovate-lanceolate 0.55–1.1 cm × 0.25–0.4 cm	lanceolate, narrowly elliptic or ovate-triangular, 0.6–0.8 cm × 0.25–0.3 cm
Flower			
-Calyx			
-Tube	campanulate, 4–5 mm long	obconic, 3–4 mm long	obconic, 3–4 mm long
-Lobe	acicular or subulate, 1.5–3 mm long	filiform-subulate,2–2.5 mm long	filiform to filiform-conical, 1.5–3 mm long
-Coroll	trumpet-shaped, 8–14 mm long, 5–8 mm in diam.	obconic 8.5–12 mm long, 5–8 mm in diam.	obconic 8–14 mm long, 5–10 mm in diam.
-Lobe	ovate to broadly ovate, 2–3 mm long	ovate-orbicular, 2.5–3 mm long	ovate, 2.5–3.5 mm long
-Plicae	triangular-ovate, with 5–10 fimbriae	ovate-oblong, with 8–10 fimbriae	ovate, with 15–20 fimbriae
-Stamen			
-Filament	filiform-subulate, 3–6 mm long	filiform, 3–3.5 mm long	filiform-subulate, 3–4 mm long
-Anther	rectangular-rounded	ellipsoid	ellipsoid
-Pistil			
-Ovary	ellipsoid or fusiform, 2.5–3 mm long	ellipsoid, 2.5–3 mm long	ellipsoid, 3–4 mm long
-Style	clavate, 0.5–1 mm long	linear, 0.7–1.5 mm long	clavate, 0.7–1.2 mm long
Fruit	obovoid, 4–7 mm long	obovoid, 4.5–5.5 mm long	obovoid, 4–5 mm long
Seed	ovate-triangular, 1–1.5 mm long	ellipsoid, 1.3–1.5 mm long	ellipsoid, 1.3–1.5 mm long

#### Additional specimens examined.

*Gentianamopanshanensis.* China. Yunnan: Xinping County, Mopan Mountain, alt. 2509 m, 13 August 2012, in flower and fruit, *Xinping County Census team 5304270757* (IMDY0019083); same location, alt. 2406 m, 18 June 2023, in flower and fruit, *T. Chen et al. XP23338* (YUKU).

*Gentianamairei*. China. Yunnan: Dali City, Cangshan Mountain, alt. 3800 m, 22 July 2009, *Z.J.Yin et al. 1631* (KUN-1220364); Jingdong County, Wuliangshan Mountain, alt. 3100 m, 19 November 1956, *B.Y.Qiu 53823* (KUN-00088281).

*Gentianapanthaica*. China. Yunnan: Heqing County, Mae Shan, 15 August 2020, *Q.P. Wang et al*. *HQ 8930* (YUKU); Nanjian County, Wuliangshan Mountain, alt. 2270 m, 24 March 2012, *E.D. Liu et al*. *3587* (KUN-1224606); Dali City, Cangshan Mountain, alt. 3800 m, 15 July 2009, *Z.J.Yin et al. 1362* (KUN-1220362); same location, 13 July 2009, *Z.J.Yin et al. 1111* (KUN-1220361); Luquan County, Daheiqing, alt. 3150 m, 2 July 1990, *R.F.Fang et al*. *83* (KUN-551847).

## Supplementary Material

XML Treatment for Gentiana mopanshanensis

## ﻿Additional information

### Author ORCIDs

Tao Chen https://orcid.org/0009-0004-1045-1662

Ting-Ting Wang https://orcid.org/0009-0000-1593-1738

Shao-Yun Liu https://orcid.org/0009-0001-6524-7632

Huan-Chong Wang https://orcid.org/0000-0001-8562-8849

### Data availability

All of the data that support the findings of this study are available in the main text.

## ﻿Appendix 1

**Table A1.** Species sequence information downloaded from the GenBank.

GenBank	Species	Voucher information	Herbarium
KU512339	*G.panthaica* Prain et Burk.	GXJ2011-055	
KT907686	*G.panthaica* Prain et Burk.	Favre & Matuszak 061a	KUN
KT907673	*G.mairei* Levl.	Wang hong et al. 01-0062	KUN
KT907643	*G.epichysantha* Hand.-Mazz.	Favre & Matuszak 131a	KUN
KF563953	*G.epichysantha* Hand.-Mazz.	Favre & Matuszak 131a	KUN
KT907728	*G.tatsienensis* Franch.	Wang Lisong, et al. 07-13	KUN
KU512348	*G.pseudoaquatica* Kusnezow	GXJ2011-043	
KT907644	*G.faucipilosa* H. Smith	Favre & Matuszak 37a	KUN
KF563954	*G.faucipilosa* H. Smith	Favre and Matuszak 37a	KUN
MT483837	*G.haynaldii* Kanitz		
MT483859	*G.haynaldii* Kanitz		
KT907613	*G.asterocalyx* Diels	Favre & Matuszak 106a	KUN
KT907653	*G.heleonastes* H. Smith ex Marq.	Favre 206a	KUN
KT907646	*G.forrestii* Marq.	Penghua, Liu Ende et al. 9538	KUN
KT907705	*G.rubicunda* Franch.	Wang hong et al. 03-1098	KUN
KU512351	*G.rubicunda* Franch.	YG2011392	
KT907651	*G.grata* H. Smith	Favre & Matuszak 32a	KUN
KT907666	*G.linoides* Franch. ex Hemsl.	Chen HS, CHC 2866	TNM
KU512331	*G.linoides* Franch. ex Hemsl.	GXJ2011-068b	
KT907687	*G.papillosa* Franck.	Chen HS, CHC 2867	TNM
KT907645	*G.flavomaculata* Hayata	Chen HS, CHC 2364	TNM
KT907618	*G.bella* Franch. ex Hemsl.	H.S. Chen CHC 2850	TNM
KT907741	*G.zollingeri* Fawcett	Chen HS CHC 2372	TNM
KU512311	*G.algida* Pall.	GXJ20130174	
KU512312	*G.algida* Pall.	PG110833	
KT907652	*G.handeliana* H. Smith	Liu Ende et al. 1209080	LZ
KT907680	*G.nubigena* Edgew.	Wang Lisong, et al. 07-55	KUN
KT907723	*G.striolata* T. N. Ho	Favre 221a	KUN
KU512317	*G.atuntsiensis* W. W. Smith	GXJ2011-072	
KU512318	*G.atuntsiensis* W. W. Smith	GXJ2011-080	
KT907627	*G.cephalantha* Franch. ex Hemsl.	Favre 325	KUN
KF563950	*G.cephalantha* Franch. ex Hemsl.	Favre 325	KUN
KT907642	*G.duclouxii* Franch.	Favre & Matuszak 076	KUN
MN124367	*G.lawrencei var. farreri* (I. B. Balfour) T. N. Ho	fu2016060_2	
MN124366	*G.lawrencei var. farreri* (I. B. Balfour) T. N. Ho	fu2016176_5	
KT907624	*G.caelestis* (Marq.) H. Smith	Favre & Matuszak 193a	KUN
KT907715	*G.sino-ornata* Balf.f.	Favre & Matuszak 224a	KUN
KU512319	*G.caelestis* (Marq.) H. Smith	GXJ2011-078	
KF563963	*G.purdomii* Marq.	Favre 311	KUN
KF563961	*G.phyllocalyx* C. B. Clarke	Favre & Matuszak 33a	KUN
KT907690	*G.phyllocalyx* C. B. Clarke	Favre & Matuszak 33a	KUN
MN339943	*G.stipitata* Edgew.		
MN339944	*G.stipitata* Edgew.		
AJ489922	*Swertiarosulata* (Baker) Klack.	Piso, Wohlhauser, Zeltner M023	NEU
MT199413	*Haleniataruga-gasso* Gilg	MO05693166	
MK416129	*Gentianellagentianoides* (Franch.) H. Smith	xuechy090094	
